# Soluble guanylate cyclase stimulators and activators: new horizons in the treatment of priapism associated with sickle cell disease

**DOI:** 10.3389/fphar.2024.1357176

**Published:** 2024-02-07

**Authors:** Dalila Andrade Pereira, Tammyris Helena Rebecchi Silveira, Fabiano Beraldi Calmasini, Fábio Henrique Silva

**Affiliations:** ^1^ Laboratory of Pharmacology, São Francisco University Medical School, Bragança Paulista, SP, Brazil; ^2^ Department of Pharmacology, Universidade Federal de São Paulo, Escola Paulista de Medicina, São Paulo, SP, Brazil

**Keywords:** anemia, corpus cavernosum, cGMP, erectile dysfunction, nitric oxide

## Abstract

Priapism, defined as a prolonged and often painful penile erection occurring without sexual stimulation or desire, is a common complication in sickle cell disease (SCD), affecting up to 48% of male patients. This condition presents significant clinical challenges and can lead to erectile dysfunction if not properly managed. Current pharmacological treatments for SCD-related priapism are primarily reactive rather than preventative, highlighting a gap in effective medical intervention strategies. A critical factor in developing priapism is the reduced basal bioavailability of nitric oxide (NO) and cyclic guanosine monophosphate (cGMP) in erectile tissues. New prevention strategies should ideally target the underlying pathophysiology of the disease. Compounds that stimulate and activate soluble guanylate cyclase (sGC) emerge as potential therapeutic candidates since these compounds have the property of inducing cGMP production by sGC. This review explores the potential of sGC stimulators and activators in treating priapism associated with SCD. We discuss the advantages of these agents in the face of the challenging pathophysiology of SCD. Additionally, the review underscores the impact of intravascular hemolysis and oxidative stress on priapism pathophysiology in SCD, areas in which sGC stimulators and activators may also have beneficial therapeutic effects.

## 1 Introduction

Sickle cell disease (SCD) is a genetic pathology affecting millions of people globally, resulting from a mutation in the β-globin gene, producing hemoglobin S (HbS) (Kato et al., 2018; Kavanagh et al., 2022). In low oxygenation, HbS polymerizes in erythrocytes, causing cellular dehydration, membrane rigidity, and formation of sickled erythrocytes, which lead to hemolysis (Kato et al., 2018). The impacts of altered morphology and compromised rheology of sickle erythrocytes are profound and varied. Patients with SCD often face painful vaso-occlusive episodes, acute chest syndrome, stroke, and chronic organ damage, drastically affecting quality and life expectancy (Kavanagh et al., 2022). The disease also causes changes in erectile function, with priapism being a common complication (Bivalacqua et al., 2022).

Priapism is characterized by prolonged penile erection in the absence of sexual stimulation or arousal (Bivalacqua et al., 2022). Ischemic priapism is characterized by reduced or absent penile blood flow, leading to painful erections (Salonia et al., 2014). Up to 48% of men with SCD have experienced some episode of priapism during their lives (Arduini and Trovó de Marqui, 2018). Recurrent episodes of priapism can cause progressive damage to erectile tissue, leading to fibrosis and, eventually, permanent erectile dysfunction (Musicki and Burnett, 2020). Studies indicate that approximately 30% of men who experience priapism end up developing erectile dysfunction, highlighting the severity of this complication (Mantadakis et al., 1999; Adeyoju et al., 2002; Alvaia et al., 2020).

Despite the high incidence of priapism, the treatment of priapism associated with SCD remains a clinical challenge (Bivalacqua et al., 2022). The main treatments available focus mainly on symptomatic relief without offering effective prevention against the occurrence of these episodes (Bivalacqua et al., 2022). The need for more effective and safer approaches is essential. Over the past 20 years, studies have reported that the reduction in basal bioavailability of nitric oxide (NO)- cyclic guanosine monophosphate (cGMP) in erectile tissue is an important factor in the development of priapism, in addition to hematological factors (Champion et al., 2005; Bivalacqua et al., 2012; Bivalacqua et al., 2013; Lagoda et al., 2013; Lagoda et al., 2013; Ning et al., 2014; Silva et al., 2016b; Musicki et al., 2018; Musicki et al., 2021; Musicki et al., 2021; Pereira et al., 2022). In this context, agents that stimulate and activate soluble guanylate cyclase (sGC) emerge as potential therapeutic candidates since these compounds have the property of inducing cGMP production by sGC (Mónica and Antunes, 2018; Sandner et al., 2021).

This review explores the potential of sGC stimulators and activators in treating priapism associated with SCD. We discuss the advantages of these agents in the face of the challenging pathophysiology of SCD. Furthermore, we highlight the implications of intravascular hemolysis and oxidative stress on the pathophysiology of priapism in SCD, areas in which sGC stimulators and activators may also have beneficial therapeutic effects.

## 2 Molecular mechanism of penile erection

**FIGURE 1:**
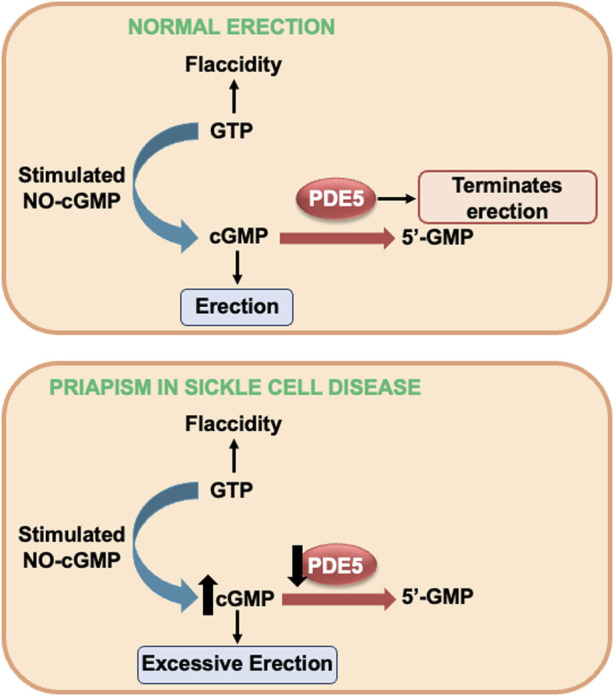
NO-cGMP-dependent mechanism of penile erection on top and alterations in NO-cGMP-PDE5 signaling in SCD-induced priapism. GMP, guanosine monophosphate (GMP) GTP, guanosine triphosphate; cGMP, cyclic GMP; NO, nitric oxide; PDE5, phosphodiesterase type 5; sGC, soluble guanylyl cyclase.

Penile tumescence is a complex process involving vascular, neural, and hormonal systems (MacDonald and Burnett, 2021). NO is the primary mediator of the erection process, being produced by the enzyme endothelial NO synthase (eNOS) present in the endothelium and by neuronal NO synthase (nNOS) present in the cavernous nerve (MacDonald and Burnett, 2021). Upon erectile stimulation, NO permeates the smooth muscle cells and binds to the ferrous iron (Fe^2+^) within the heme domain of sGC. This binding stimulates sGC to synthesize the secondary messenger, cGMP (Andersson, 2011). High cGMP concentrations activate cGMP-dependent protein kinase (PKG), which relaxes cavernous smooth muscles and penile vessels, culminating in erection (Andersson, 2011) (Figure 1).

Detumescence is the process of the penis returning to its flaccid state. At this stage, the enzyme phosphodiesterase type 5 (PDE5) plays a central role by converting cGMP into guanosine monophosphate (GMP), thus ending the erection (Andersson, 2011; MacDonald and Burnett, 2021) (Figure 1). This cGMP production and degradation cycle, orchestrated by sGC and PDE5, respectively, is essential to maintain the balance between tumescence and detumescence. Dysfunction in this mechanism can result in disorders such as erectile dysfunction or priapism, underlining the importance of this balance in normal penile physiology (MacDonald and Burnett, 2021).

## 3 An overview of the effects of sGC stimulators and activators in erectile dysfunction models

In the 1990s, the development of YC-1 marked a significant advance as the first sGC stimulator that acts independently of NO. This pioneering advance stimulated the development of new, more potent, and selective compounds capable of directly stimulating sGC for cGMP synthesis (Sandner et al., 2021). Subsequent advances in the area led to the differentiation of these molecules into two categories: sGC stimulators and activators (Mónica and Antunes, 2018; Sandner et al., 2021).

sGC stimulators, such as YC-1, BAY 41-2272, BAY 63-2521, BAY 41-8543, and BAY 60-4552, act independently of NO, but their effectiveness depends on the presence of the heme group in sGC. They activate sGC and increase the sensitivity of enzyme to NO, elevating cGMP levels (Stasch et al., 2001; Priviero et al., 2005; Gur et al., 2010). sGC activators, such as BAY 58-2667, BAY 60-2770, and HMR-1766, have different mechanisms of action and therapeutic potentials (Sandner et al., 2021). *In vitro* studies revealed that sGC can exist in oxidized states or without the heme group (Stasch et al., 2006; Sandner et al., 2021). sGC activators are particularly promising due to their property of activating sGC in conditions where the iron in the heme group is oxidized (sGC-Fe^3+)^ or in the absence of the heme group. This aspect is crucial, as heme (Fe^3+^) oxidation can make sGC insensitive to endogenous NO, inhibiting the activation of the sGC-cGMP-PKG pathway in several tissues (Stasch et al., 2006; Sandner et al., 2021).

Over the last 20 years, several experimental studies have evaluated the impact of sGC stimulators on erectile function. BAY 41-2272, for example, induces penile erection in rabbits, an effect that is enhanced by the administration of sodium nitroprusside (SNP), an exogenous NO donor (Bischoff et al., 2003). Furthermore, it was observed that BAY 41-8543, another sGC stimulator, when administered intracamerally, increases intracavernous pressure (ICP) and acts synergistically with the SNP (Lasker et al., 2013). BAY 41-2272 also promotes relaxation of the corpora cavernosa of rabbits, rats, and humans (Baracat et al., 2003; Silva et al., 2016b) and reverses erectile and ejaculatory dysfunction in rats under chronic NO deficiency (Claudino et al., 2011; da Silva et al., 2012). In diabetic or eNOS-deficient mice, BAY 41-2272 increases relaxation induced by activation of the NO-cGMP pathway (Nunes et al., 2015). A recent study highlighted that riociguat (BAY 63-2521) promotes concentration-dependent relaxation in the corpora cavernosa of mice (Olivencia et al., 2023). Notably, in 2013, the approved riociguat for treating pulmonary hypertension, underlining its therapeutic potential in conditions of low cGMP bioavailability.

Regarding sGC activators, cinaciguat (BAY 58–2667) induces concentration-dependent relaxation in corpora cavernosa of mice, accompanied by an increase in cGMP production (Decaluwé et al., 2017; de Oliveira et al., 2023; Olivencia et al., 2023). Furthermore, BAY 60-2770 promotes concentration-dependent relaxation in rabbit corpora cavernosa and exhibits potent erectile activity in rats (Lasker et al., 2013). Preclinical research has shown that treatment with BAY 60-2770 reverses erectile dysfunction, normalizes changes in the NO-cGMP pathway in the corpora cavernosa, and increases cGMP levels in erectile tissue in obese mice treated with a high-fat diet, as well as reversing voiding dysfunction (Alexandre et al., 2014; Leiria et al., 2014; Silva et al., 2014).

sGC stimulators and activators also affect oxidative stress, an imbalance between the production of reactive oxygen species (ROS) and the antioxidant capacity of body (Alexandre et al., 2014; Leiria et al., 2014; Silva et al., 2014; Nunes et al., 2015). One of the main consequences of oxidative stress is the increase in superoxide anion (O_2_^−^), which reacts quickly with NO, thus reducing its bioavailability (Pacher et al., 2007). Studies demonstrate that BAY 41-2272 is effective in reducing the production of superoxide anion and decreasing the expression of NADPH oxidase subunits, a source of superoxide anion, in the corpora cavernosa of mice, through a cGMP-dependent mechanism (Teixeira et al., 2007). In corpora cavernosa of diabetic mice, BAY 41-2272 reduced the excessive production of superoxide anion and the expression of NADPH oxidase subunits (Nunes et al., 2015). Similarly, BAY 60-277 also reduced excessive ROS production in the corpora cavernosa of obese mice (Silva et al., 2014).

In summary, sGC stimulators and activators are compounds capable of increasing the bioavailability of cGMP in the corpora cavernosa through the activation of sGC and indirectly by reducing the production of superoxide anion. These actions contribute significantly to the normalization of erectile function and the reduction of oxidative stress. These properties highlight the therapeutic potential of these compounds, particularly in conditions where cGMP bioavailability in erectile tissue is compromised.

## 4 Effect of sGC stimulators and activators on sickle cell disease

SCD presents several clinical complications that are associated with dysfunction of the NO-cGMP pathway, including pulmonary hypertension and vaso-occlusive events, which significantly impact patients’ quality of life (Kavanagh et al., 2022). Recently, attention has focused on the therapeutic potential of sGC stimulators and activators in this context. These compounds represent a new approach to the treatment of SCD, focusing not only on improving symptoms but also on modulating the underlying pathophysiological pathways (Potoka et al., 2018; Ferreira et al., 2020; Sandner et al., 2021; Tchernychev et al., 2021).

An experimental study investigated the effect of treatment with the sGC stimulator (BAY 41-8543) and the sGC activator (BAY 54-6544) in mice with SCD(Potoka et al., 2018). The results revealed that acute treatment with BAY 54-6544 was more effective than BAY 41-8543 in improving pulmonary artery endothelial function. This finding suggests that sGC in the pulmonary arteries of SCD transgenic mice is in an oxidized state (Potoka et al., 2018). Furthermore, chronic treatment with BAY 54-6544 also reversed pulmonary hypertension without impacting systemic blood pressure in the animals (Potoka et al., 2018).

A recent study highlighted that both the sGC activator, BAY 60-2770, and the sGC stimulator, BAY 41-2272, reduce these vaso-occlusive events in mice with SCD. This effect occurs mainly by decreasing the recruitment of leukocytes to the endothelium, thus reducing vascular occlusion (Ferreira et al., 2020). Similarly, olinciguat, another sGC stimulator, was shown to reduce inflammation, vaso-occlusion, and nephropathy in a murine model for sickle cell anemia, highlighting the therapeutic potential of these compounds (Tchernychev et al., 2021). Interestingly, BAY 41-2272, but not BAY 60-2770, demonstrated the ability to increase γ-globin gene expression and fetal hemoglobin production in K562 erythroleukemic cell cultures, offering an additional perspective on SCD therapy (Ferreira et al., 2020).

In SCD, characterized by intravascular hemolysis, both exogenous and endogenous NO are neutralized by free hemoglobin in the plasma or interstitial space. Research indicates that blood flow responses to infusions of NO donor treatments in humans and mice with SCD are impaired (Nath et al., 2000; Reiter et al., 2002; Kaul et al., 2004; Hsu et al., 2007). However, intravascular hemoglobin does not affect vasodilation mediated by sGC stimulators and activators, such as BAY 41-8543 and BAY 60-2770 (Potoka et al., 2018). These results suggest a significant clinical advantage of these compounds over NO donors, especially in contexts where NO inactivation is a concern, such as SCD.

## 5 NO-cGMP-PDE5 pathway dysfunction in SCD-associated priapism pathophysiology

In recent years, experimental evidence has shown that priapism associated with SCD occurs mainly due to a failure in the penile detumescence mechanism, together with hematological changes. The change in this mechanism begins with a reduction in the basal bioavailability of NO of endothelial origin in the corpora cavernosa, leading to a subsequent decrease in PDE5 function (Champion et al., 2005; Bivalacqua et al., 2013; Lagoda et al., 2013; Lagoda et al., 2014; Ning et al., 2014; Silva et al., 2016b; Musicki et al., 2018; Musicki and Burnett, 2020; Pereira et al., 2022).

Studies carried out in transgenic mice for SCD, mice deficient for eNOS, and mice deficient for eNOS and nNOS showed that these animals present increased erectile response when subjected to electrical stimulation of the cavernous nerve, together with fibrotic changes in the penis (Burnett et al., 2002; Champion et al., 2005; Bivalacqua et al., 2009; Silva et al., 2016b; Pinheiro et al., 2022). SCD mice exhibit a priapism phenotype with an augmented relaxation response of the corpus cavernosum *in vitro*, triggered by activation of the NO-sGC pathway (Claudino et al., 2009). Heightened penile erection and increased relaxation of the corpus cavernosum smooth muscle are linked to decreased PDE5 protein expression in penile tissue from SCD mice (Champion et al., 2005; Bivalacqua et al., 2013; Silva et al., 2016a; Silva et al., 2016b; Musicki et al., 2018; Musicki et al., 2021; Pinheiro et al., 2022). Thus, when an erectile stimulus occurs *in vivo*, cGMP accumulates in cavernous smooth muscle cells, resulting in excessive and prolonged penile vascular dilation (i.e., priapism), as cGMP is not degraded as a consequence of PDE5 dysregulation (Musicki and Burnett, 2020) (Figure 1).

PDE5 expression is positively regulated by basal cGMP levels in the penis (Corbin et al., 2000; Lin et al., 2002). The penis of SCD transgenic mice has lower basal cGMP levels due to lower eNOS activity, which is attributed to reduced phosphorylation of eNOS at the SER-1177 site, uncoupling of eNOS, and decreased binding of eNOS to the Heat shock protein 90 (HSP90) (Musicki et al., 2011; Musicki et al., 2012; Bivalacqua et al., 2013; Musicki et al., 2014; Sopko et al., 2015; Silva et al., 2016b). Similarly, penile tissue samples from men with SCD who experience priapism also demonstrate a decrease in eNOS and PDE5 protein expression, reinforcing the correlation between eNOS activity, cGMP levels, and PDE5 regulation (Lagoda et al., 2013).

Oxidative stress is another factor that participates in the pathophysiology of priapism in SCD and contributes to the reduction of basal NO bioavailability. Studies have shown that the penis of mice with SCD exhibits increased production of ROS originating from several sources (Musicki et al., 2012; Musicki et al., 2014; Silva et al., 2016b; Pereira et al., 2022). One of these sources is NADPH oxidase, an enzyme complex that facilitates the transfer of electrons from cytosolic NADPH to molecular oxygen, significantly contributing to superoxide production (Zhang et al., 2020). Furthermore, another source is the elevated activity of xanthine oxidase, an enzyme that catalyzes the conversion of hypoxanthine and xanthine into uric acid, generating superoxide as a byproduct (Aslan et al., 2001; Bivalacqua et al., 2013). Another relevant source is the uncoupled form of eNOS, which generates superoxide instead of producing NO (Bivalacqua et al., 2013). This complex interaction between the diverse sources of ROS and the reduction in NO availability underlines the importance of oxidative stress in the pathogenesis of priapism in patients with SCD, offering possible targets for therapeutic interventions.

Intravascular hemolysis is another critical change contributing to the reduction in NO bioavailability and, consequently, to the development of priapism (Morris et al., 2005; Nolan et al., 2005; Cita et al., 2016). The rupture of red blood cells within blood vessels releases hemoglobin, arginase, and other cellular contents into the circulation (Morris et al., 2005). Free hemoglobin (HbFe^2+^) in plasma or interstitial spaces reacts rapidly with NO, consequently reducing NO bioavailability (Reiter et al., 2002; Schaer et al., 2013). Furthermore, an additional element that reduces NO bioavailability is the increased activity of plasma arginase, which metabolizes L-Arginine, the primary substrate for NO synthesis (Morris et al., 2005). A recent study showed that induction of intravascular hemolysis in healthy mice resulted in increased cavernous relaxation induced by cholinergic and nitrergic stimulation in the corpus cavernosum, similar to that observed in SCD mice (Iacopucci et al., 2022).

Hydroxyurea was the first drug approved by the FDA to treat patients with SCD (Steinberg et al., 2003). A single dose of hydroxyurea in patients with SCD elevates plasma nitrate and nitrite levels, indicating that hydroxyurea can generate intravascular NO (Glover et al., 1999). Despite being widely used by male patients with SCD, few studies have reported the beneficial effects of hydroxyurea on priapism (Saad et al., 2004; Anele et al., 2014). The compound RVT-FxMe is a new compound that has NO donating property. Recent preclinical studies evaluated the effect of treatment with hydroxyurea or RVT-FxMe, intending to normalize the bioavailability of NO-cGMP in erectile tissue and reverse the priapism phenotype in SCD mice (Pinheiro et al., 2022). However, 2 weeks of treatment with RVT-FxMe or hydroxyurea did not modify the priapism phenotype (Pinheiro et al., 2022; Pereira et al., 2023). It is likely that the NO generated by hydroxyurea and RVT-FxMe are being inactivated by plasma hemoglobin or ROS before binding to sGC in the smooth muscle cells of the corpus cavernosum (Pereira et al., 2023).

## 6 sGC stimulators and activators as drug candidates for the treatment of priapism in SCD

Ideally, new prevention strategies should act on the pathophysiological basis of the disease. As mentioned above, reduced cGMP bioavailability and reduced PDE5 expression and activity are important changes contributing to the development of priapism in SCD (Musicki and Burnett, 2020). Preclinical studies showed that compounds that were able to normalize cGMP bioavailability and reduce ROS production in the erectile tissue of SCD mice were effective in reversing the priapism phenotype, mainly through normalizing PDE5 expression in the penis (Bivalacqua et al., 2013; Silva et al., 2016b; Musicki et al., 2018; Musicki et al., 2021; Pereira et al., 2022).

**FIGURE 2:**
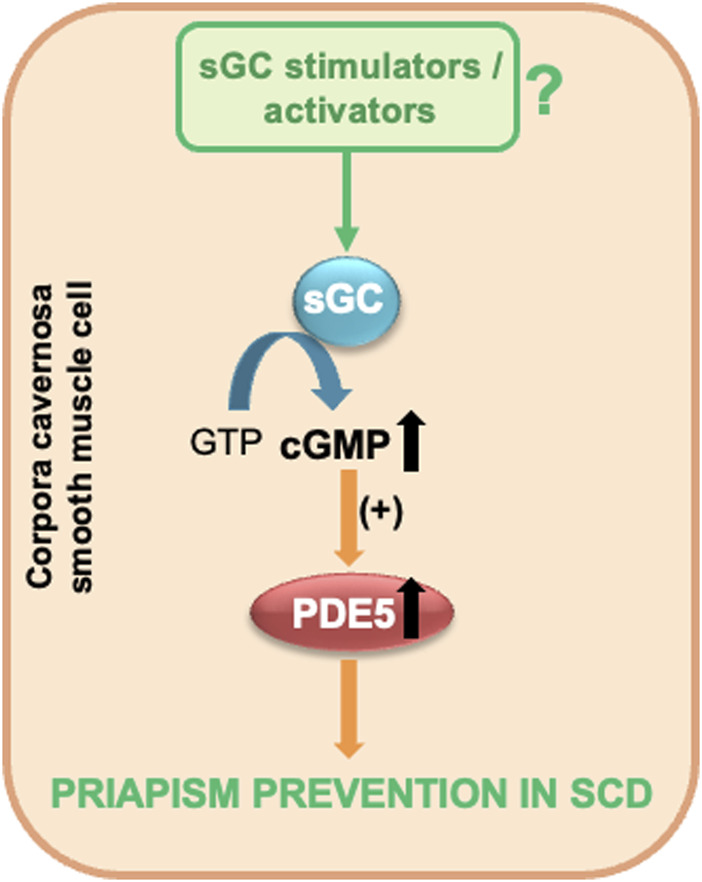
sGC stimulators and activators as potential therapeutic candidates for priapism prevention in SCD. GTP, guanosine triphosphate; cGMP, cyclic guanosine monophosphate; PDE5, phosphodiesterase type 5; sGC, soluble guanylyl cyclase.

Considering the experimental evidence presented, sGC activators and stimulators emerge as promising therapeutic options in the management of priapism associated with SCD. These agents can potentially elevate basal cGMP levels in the corpora cavernosa, which may, in turn, normalize PDE5 activity and expression. This approach offers an effective strategy that can prevent the development of priapism (Figure 2). sGC protein expression is not modified in mouse erectile tissue (Champion et al., 2005). However, it is necessary to highlight the need for further studies to investigate the protein expression of sGC in the erectile tissue of men with SCD. Furthermore, determining the redox state of sGC is essential to establish the most suitable class.

Unlike NO-donating compounds, sGC stimulators and activators are not inactivated by plasma hemoglobin or excess ROS. This characteristic gives them a significant therapeutic advantage, especially in pathological conditions where these factors interfere with the bioavailability of NO. Furthermore, an additional advantage of sGC stimulators and activators is their ability to reduce superoxide anion production (Teixeira et al., 2007; Silva et al., 2014; Nunes et al., 2015). This effect helps mitigate the inactivation of endogenous NO, thus contributing to the preservation of normal vascular function and preventing disorders related to endothelial dysfunction.

A critical aspect of treating priapism in SCD includes preventing recurrent episodes of priapism and managing acute episodes of priapism. sGC stimulators and activators are promising for prevention. The efficacy of these agents in elevating basal cGMP levels in corpora cavernosa offers a promising preventive strategy against the development of priapism episodes. However, it is essential to highlight that for immediate relief during acute priapism episodes, other pharmacological or medical interventions may be required. This bifurcated approach, preventive and acute management, highlights the complexity of treating priapism in SCD and underscores the need for comprehensive research to optimize treatment strategies.

## 7 Conclusion

Priapism associated with SCD represents a significant clinical challenge, requiring therapeutic approaches that go beyond symptomatic treatment. Accumulating evidence indicates that NO-cGMP pathway dysfunction is a central component in the pathogenesis of priapism in SCD, highlighting the need for strategies to address this dysregulation. In this context, sGC stimulators and activators emerge as promising compounds in the preventive treatment of priapism in patients with SCD. These compounds have demonstrated efficacy in normalizing cGMP levels and attenuating oxidative stress, thus addressing components of the pathophysiological basis of priapism. Due to the complex pathophysiology of priapism, the future treatment of priapism in SCD may involve combined and selective therapeutic approaches, combining different treatment modalities rather than being restricted to monotherapy. This approach could optimize therapeutic outcomes by adjusting to individual patient needs and more effectively addressing the complexities of SCD.
